# Performance Characterization and Antibacterial Activity of a Composite Hydrogel Composed of Oxidized κ-Carrageenan, Acrylamide, and Silver-Based Metal–Organic Frameworks

**DOI:** 10.3390/gels11060407

**Published:** 2025-05-29

**Authors:** Bo Qi, Zhaoyu Li, Chuang Pan, Yongqiang Zhao, Xiaoshan Long, Chunsheng Li, Yueqi Wang, Xiao Hu, Di Wang, Shaoling Yang

**Affiliations:** 1 South China Sea Fisheries Research Institute, Chinese Academy of Fishery Sciences, Guangzhou 510300, China; qibo780210@163.com (B.Q.); lzy1363224884@163.com (Z.L.); silverpfoxc@hotmail.com (C.P.); zhaoyq@scsfri.ac.cn (Y.Z.); longxs2023@163.com (X.L.); lichunsheng@scsfri.ac.cn (C.L.); wangyueqi@scsfri.ac.cn (Y.W.); hnhuxiao@163.com (X.H.); wangdi@scsfri.ac.cn (D.W.); 2 Key Laboratory of Aquatic Product Processing, Ministry of Agriculture and Rural Affairs, Guangzhou 510300, China

**Keywords:** hydrogel, oxidized κ-carrageenan, Ag-MOFs, characterization, antibacterial property, biocompatibility

## Abstract

To advance seaweed polysaccharide applications in hydrogel wound dressings, five antibacterial composite hydrogels (groups A~E) were synthesized using oxidized κ-carrageenan (OKC), acrylamide (AM), and progressively increasing concentrations of silver-based metal–organic frameworks (Ag-MOFs). Systematic characterization revealed concentration-dependent effects: (1) positive correlations were obtained for the moisture content (MC, maximized at 82.70% in E) and antibacterial efficacy (dose-dependent enhancement); (2) negative impacts were obtained for the swelling ratio (SR, E: 479% vs. A: 808%); and (3) high-dose drawbacks but low-dose benefits in terms of water resistance (WR), tensile strength (TS), elongation at break (EB), and microstructure were obtained. Group B demonstrated optimal Ag-MOFs loading, enhancing TS and EB, while excessive Ag-MOFs loading in C~E significantly degraded them (*p* < 0.05). Microstructural analysis showed severe 3D spatial damage in D~E. Furthermore, cytocompatibility assessments revealed that all groups maintained a cell viability exceeding 90%, demonstrating excellent biocompatibility. Among them, A~C showed a viability statistically equivalent to the control (*p* > 0.05) and were significantly higher than D~E (*p* < 0.05). In conclusion, group B emerged as the optimal Ag-MOFs formulation and exhibited superior WR, enhanced mechanical strength (TS and EB), and potent antibacterial activity while maintaining microstructural integrity and excellent biosafety. This Ag-MOFs/OKC/PAM hydrogel provides dual infection prevention and tissue support, maximizing seaweed polysaccharide benefits with excellent biocompatibility.

## 1. Introduction

In recent years, research on seaweed-based hydrogels has predominantly concentrated on polysaccharide polymers such as agar and alginate, leading to the successful development of numerous commercial products for the medical, cosmetic, and other industries. However, despite being another seaweed polysaccharide with high production volume, abundant resources, and excellent gelling properties, carrageenan remains relatively rare in the market for hydrogel-based products. Carrageenan is a natural polysaccharide extracted from red algae and composed of galactose and dehydrated galactose, existing in the form of calcium, potassium, sodium, and ammonium salt of polysaccharide sulfuric ester. Among its different types, κ-carrageenan stands out due to its thermoreversible gelation when combined with potassium ions, as well as its strong gelling ability, making it a subject of significant scientific interest [1,2].

Carrageenan has gained widespread application in the food and the pharmaceutical industry due to its water retention, emulsification, and gelation properties. Its safety and efficacy have been globally acknowledged, as demonstrated by its inclusion in the latest editions of the U.S. Pharmacopoeia (USP-NF 2024), European Pharmacopoeia (EP 11.0), and British Pharmacopoeia (BP 2024). Moreover, as a polymer compound, carrageenan excels in forming a three-dimensional (3D) network structure, allowing for the production of hydrogels with versatile shapes, tunable mechanical strength, high water absorption capacity, and outstanding biocompatibility. These properties make it suitable not only for use as a pharmaceutical excipient in medical applications such as wound dressings, drug delivery systems, and tissue engineering scaffolds but also for other high-value medical uses [3,4].

The limited solubility of carrageenan results in single-component hydrogels that are excessively soft, brittle, and lacking in toughness, which restricts their potential applications in medical dressings. To address this, modifying carrageenan and its hydrogel systems, through chemical modification, blending with other materials, or introducing functional groups, can enhance their physicochemical properties and performance in medical applications. For instance, Zhao et al. [5] prepared a series of agar/κ-carrageenan mixed hydrogels with different mass ratios and found that κ-carrageenan had the potential to improve the physicochemical properties and drug release performance of agar hydrogels. The addition of κ-carrageenan also enhanced the drug-loading efficiency and sustained-release capacity. Ulagesan et al. [6] developed a composite hydrogel using natural polysaccharide κ-carrageenan and phycobiliprotein r-phycoerythrin from *Pyropia yezoensis*, which exhibited dose-dependent antioxidant activity and significant antimicrobial activity at a 100 µg/mL concentration. Gubaidullin et al. [7] reinforced a κ-carrageenan–gelatin hydrogel system by embedding carbon nanotubes as a structural supplementary template for the 3D net formation, which significantly improved its mechanical strength. Pahnavar et al. [8] prepared a novel double-network hydrogel based on a blend of modified κ-carrageenan and polyvinyl alcohol, demonstrating excellent biocompatibility. Additionally, polyacrylamide (PAM), a synthetic polymer known for its high water absorption capacity, tunable cross-linking properties, and biocompatibility, is widely used in advanced composite hydrogels. Its unique affinity for polysaccharides (e.g., κ-carrageenan, chitosan), proteins, and other biomolecules enables synergistic interactions that enhance both the structural integrity and functionality of hydrogel [9,10,11].

The physicochemical properties and application functions of κ-carrageenan-based hydrogel can be significantly enhanced through modification, functional group incorporation, and composite formation. In this study, κ-carrageenan was oxidatively modified using a method established in our prior research [11] to improve its physicochemical characteristics. The oxidized κ-carrageenan (OKC) was then blended with acrylamide in an appropriate proportion to prepare an OKC-based hydrogel. Finally, to endow the hydrogel with antibacterial effects, a silver-based metal–organic framework (Ag-MOFs), a type of functional nanomaterial recognized for its low toxicity, high biocompatibility, and potent antibacterial activity, was incorporated into the composite hydrogel matrix. The resulting hydrogel was systematically evaluated for its physicochemical performance, antibacterial efficacy, and cellular biocompatibility.

## 2. Results and Discussion

### 2.1. Characterization of Ag-MOFs

**Figure 1:**
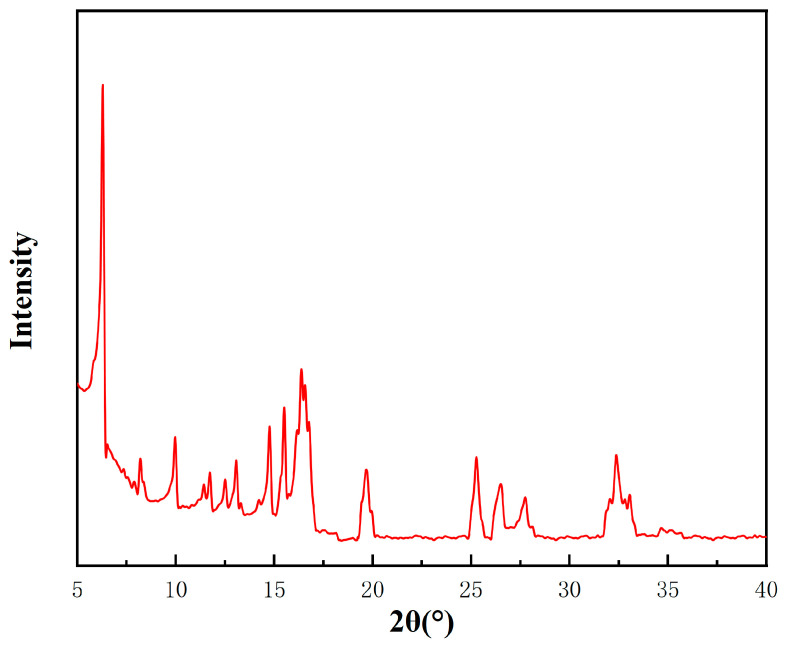
XRD pattern of Ag-MOFs.

The X-ray diffraction (XRD) pattern of the Ag-MOFs (Figure 1) showed prominent peaks at 2θ = 6.3°, 10.0°, 11.7°, 12.5°, 13.1°, 14.7°, 15.5°, 16.4°, 19.6°, 25.3°, 26.4°, 27.8°, and 32.4°, which match well with the simulated pattern reported by Lu et al. [12]. The sharp diffraction peaks indicated high crystallinity, and the baseline fluctuation between 5~15° suggested the presence of minor impurities.

**Figure 2:**
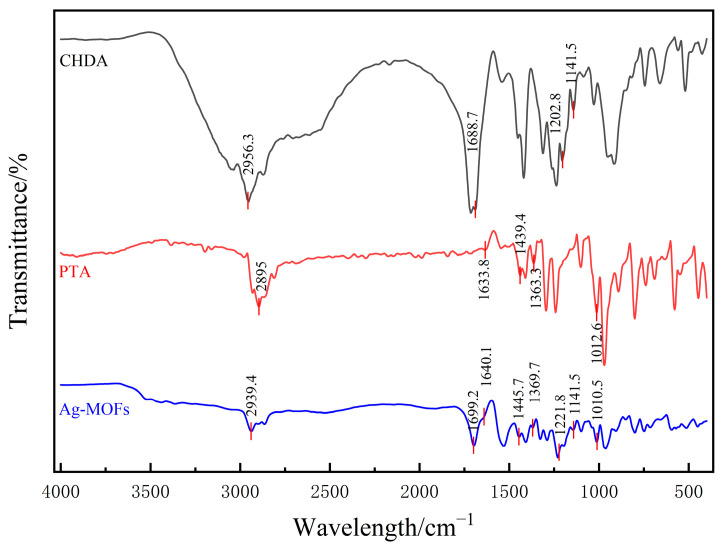
Infrared spectra of Ag-MOFs and their main synthetic materials.

The comparative FTIR analysis of the Ag-MOFs with their constituent components (CHDA and PTA) revealed significant spectral changes that confirmed successful framework formation (Figure 2). The following observations were made: (1) The broad O-H stretching vibration of carboxylic acid (~2500~3000 cm^−1^) in CHDA disappeared in the Ag-MOFs, being replaced by new carboxylate vibration peaks at 1530 cm^−1^ and 1408 cm^−1^. This transformation confirmed the deprotonation of CHDA’s carboxylic groups and their subsequent coordination with Ag^+^ ions (COO^−^-Ag^+^), which is characteristic of metal–carboxylate bond formation in MOFs. (2) The Ag-MOFs exhibited distinct new peaks in the low-frequency region (around 600 cm^−1^) that were absent in both the CHDA and PTA, which may be due to the coordination bonds formed between Ag^+^ and the carboxyl oxygen atom (from CHDA) or the phosphate oxygen atom (from PTA) (Ag-O vibration), which is a characteristic of the formation of metal–organic frameworks in MOFs.

These changes demonstrated the successful synthesis of the Ag-MOFs, whose structure incorporated both the carboxylic groups of CHDA and the phosphate groups of PTA, forming a porous coordination polymer through Ag^+^ bridging.

### 2.2. Physicochemical Properties of Ag-MOFs/OKC/PAM Hydrogel

#### 2.2.1. Moisture Content and Water Solubility

**Figure 3:**
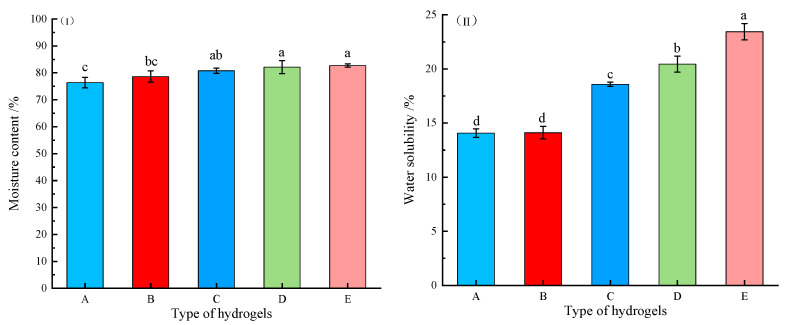
Moisture content (**I**) and water solubility (**II**) of different Ag-MOFs/OKC/PAM hydrogels. Note: Different lowercase letters (a~d) above bars in the same chart denote significant differences among groups (*p* < 0.05). Letters A~E correspond to test groups A~E, respectively.

Moisture content (MC) and water solubility (WS) are critical parameters for evaluating hydrogel’s suitability as wound dressings. Optimal wound care hydrogels should possess high moisture retention capacity to maintain a moist wound environment conducive to healing while minimizing scar formation. Conversely, WS serves as an important indicator of a hydrogel’s water resistance, where lower values correspond to better performance. The MC and WS of the OKC-based composite hydrogels with varying quantities of Ag-MOFs (Ag-MOFs/OKC/PAM) are shown in Figure 3.

As shown in Figure 3I, all five groups of Ag-MOFs/OKC/PAM hydrogels exhibited MC values exceeding normal skin (~70%) [13], satisfying the requirements for a moist wound-healing environment. Furthermore, the MC demonstrated a concentration-dependent increase with higher Ag-MOF loading, suggesting that the Ag-MOF incorporation enhanced the water-holding capacity of the Ag-MOFs/OKC/PAM to some extent. Among the five groups, the Ag-MOFs-free control group (A) showed the lowest MC (76.36%), while the highest Ag-MOFs concentration group (E) achieved the highest MC (82.7%). Notably, the statistical analysis indicated significant differences (*p* < 0.05) in MC between groups C~E compared to group A, though no significant variations were observed among groups C~E themselves, and starting from group C, the rate of MC increase slowed. This all indicated that this concentration of Ag-MOFs in group C represents a critical threshold for the effects on the MC increase rate, and beyond this point, further changes in the Ag-MOFs content had a diminished impact.

Figure 3II demonstrates a similar concentration-dependent trend for WS, with group C again serving as a pivotal point for rate changes. However, the impact of the Ag-MOFs content on WS was more significant (*p* < 0.05) than on MC. This distinction led to a markedly higher change rate in WS, particularly for the three groups with a higher Ag-MOFs content (C~E). Since WS was inversely correlated with water resistance, these results indicated that while the addition of an appropriate amount of Ag-MOFs maintained acceptable WS levels, excessive incorporation could impair water resistance. This highlights the necessity for careful optimization of the Ag-MOFs content to balance moisture retention and water resistance properties in wound dressing applications.

#### 2.2.2. Swelling Ratio

**Figure 4:**
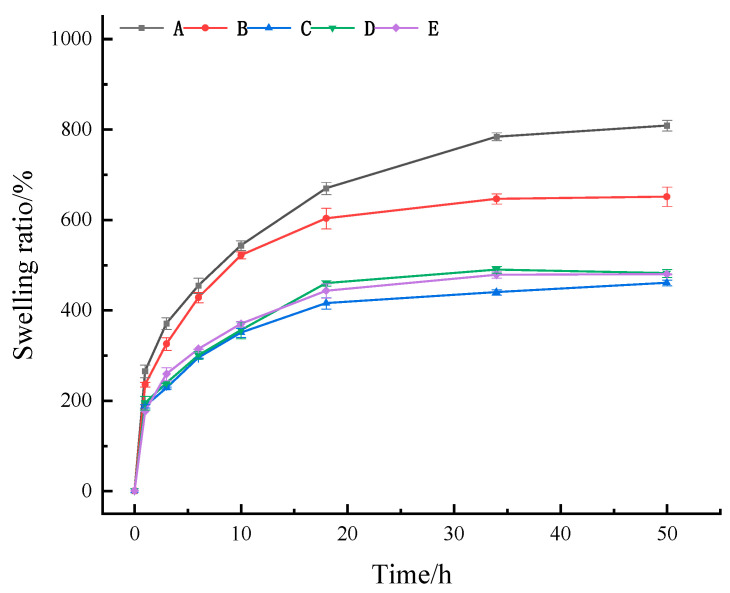
Swelling ratio of different Ag-MOFs/OKC/PAM hydrogels. Note: Letters A~E correspond to test groups A~E, respectively.

When a wound exists on the skin, it often has an exudate. If this exudate is not effectively absorbed, it can hinder the healing process. Therefore, an effective medical skin dressing must possess strong swelling capacity and the ability to absorb wound exudate efficiently. As shown in Figure 4, all five hydrogels demonstrated significant water absorption capacity within 18 h, but different types of hydrogels had different water absorption abilities. As time progressed, the swelling ratio (SR) of these five groups continued increasing, but its change rate gradually slowed. Among the four hydrogels containing Ag-MOFs (groups B~E), the SR reached equilibrium by 18 h, whereas the Ag-MOFs-free hydrogel (group A) achieved equilibrium at 34 h. During the initial 10 h period, group B (with the lowest Ag-MOFs content) showed an SR nearly equivalent to, though slightly lower than, group A. However, this difference became more pronounced after 20 h. In contrast, groups C~E (with relatively higher Ag-MOFs contents) maintained similar SR values throughout the entire experimental period, consistently demonstrating significantly lower SR values compared to groups A and B from the first hour onward. At the 50 h mark, the SR ranking was A > B > E > D > C. It was obvious that all four Ag-MOFs-containing hydrogels showed significantly lower SR than hydrogel A (808%) (*p* < 0.05), which contained no Ag-MOFs. Specifically, the SR of group B (651%) was not only significantly lower than that of A but also significantly higher than that of C (460%), D (481%), and E (479%) (*p* < 0.05). This result indicates that the incorporation of excessive Ag-MOFs negatively impacted the swelling capacity of the Ag-MOFs/OKC/PAM hydrogels. In contrast, minimal Ag-MOFs incorporation had a limited negative effect on the swelling performance.

#### 2.2.3. Mechanical Property

**Figure 5:**
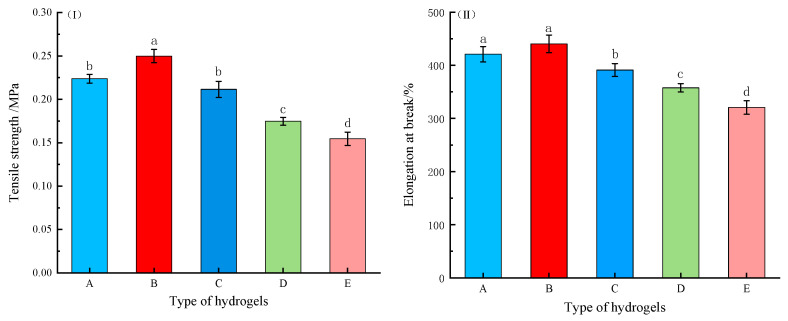
Mechanical properties of different Ag-MOFs/OKC/PAM hydrogels: (**I**) tensile strength; (**II**) elongation at break. Note: Different lowercase letters (a~d) above bars in the same chart denote significant differences among groups (*p* < 0.05). Letters A~E correspond to test groups A~E, respectively.

Medical dressings must demonstrate adequate stress resistance under complex physiological conditions. Previous studies have confirmed that oxidative modification can significantly improve the mechanical properties of κ-carrageenan-based hydrogels [11]. To evaluate the mechanical performance of the Ag-MOFs/OKC/PAM hydrogels, tensile strength (TS) and elongation at break (EB) were both measured. As shown in Figure 5I,II, Ag-MOFs incorporation significantly affected both TS and EB of these composite hydrogels (*p* < 0.05). Both parameters followed similar trends, initially increasing and then decreasing as the Ag-MOFs content increased. Specifically, group B (low Ag-MOFs content) exhibited higher TS (0.249 MPa) and EB (440%) compared to group A (0.223 MPa and 421%, respectively), which contained no Ag-MOFs. In contrast, the other three groups (C, D, and E), with higher Ag-MOF contents, showed lower TS and EB values than group A. Statistical analysis revealed significant differences in TS between group A and groups B, D, and E, as well as significant differences in EB between group A and groups C, D, and E. These results demonstrate that while moderate Ag-MOFs addition (as in group B) improved the mechanical strength of the Ag-MOFs/OKC/PAM hydrogels, excessive incorporation negatively impacted the hydrogel performance.

### 2.3. Scanning Electron Microscopy

**Figure 6:**
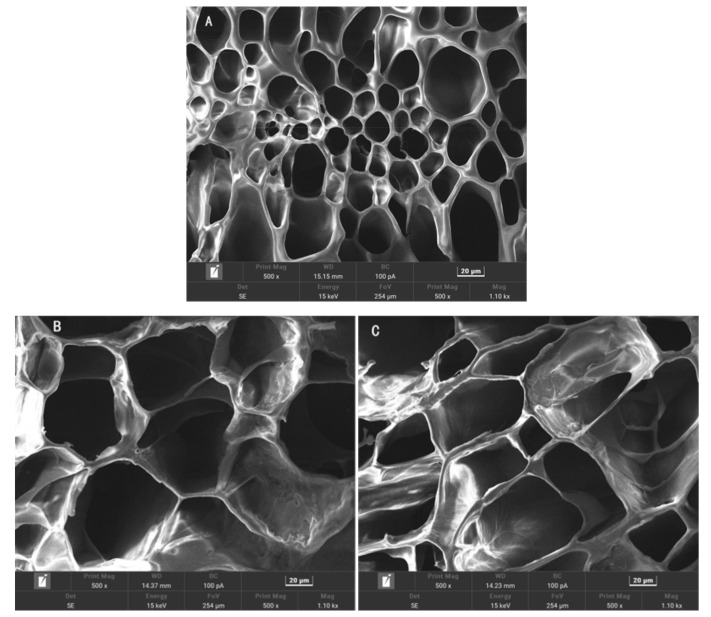

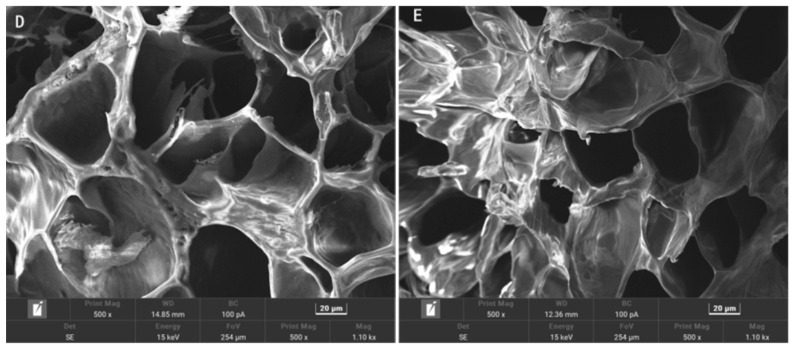
SEM images of different Ag-MOFs/OKC/PAM hydrogels. Note: Letters (**A**–**E**) correspond to test groups A~E, respectively.

The microstructural impact of the incorporation of Ag-MOFs in the OKC-based composite hydrogels was investigated by scanning electron microscopy (SEM), as depicted in Figure 6.

The SEM images revealed three key findings: (1) The Ag-MOF-free hydrogel (A) exhibited numerous small pores with thick, smooth walls but poor pore size distribution uniformity. (2) Compared to hydrogel A, the Ag-MOF-containing hydrogels (B–E) exhibited larger average pore diameters, thinner pore walls, and improved pore size distribution uniformity. This structural modification facilitates enhanced water retention and drug-loading capacity, which can enhance the hydrogel’s functionality. (3) Among groups B to E, increasing Ag-MOFs loading progressively compromised the 3D pore structure integrity. Notably, groups B and C maintained relatively intact architectures similar to group A. Groups D and E showed significant structural collapse.

These findings suggest that Ag-MOFs incorporation significantly altered the microstructure of the Ag-MOFs/OKC/PAM hydrogels, with the loading concentration critically determining structural preservation. Higher Ag-MOFs contents led to greater disruption of the pore architecture. An optimal Ag-MOF content can thereby tailor hydrogel’s properties for specific biomedical applications such as antibacterial wound dressings or controlled drug delivery systems.

### 2.4. Antimicrobial Activity

**Figure 7:**
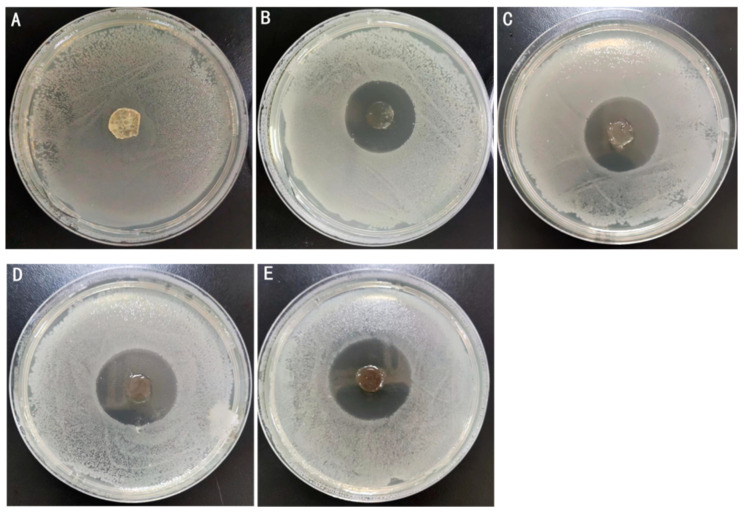
Inhibition test against *Escherichia coli*. Note: Letters (**A**–**E**) correspond to test groups A~E, respectively.

**Figure 8:**
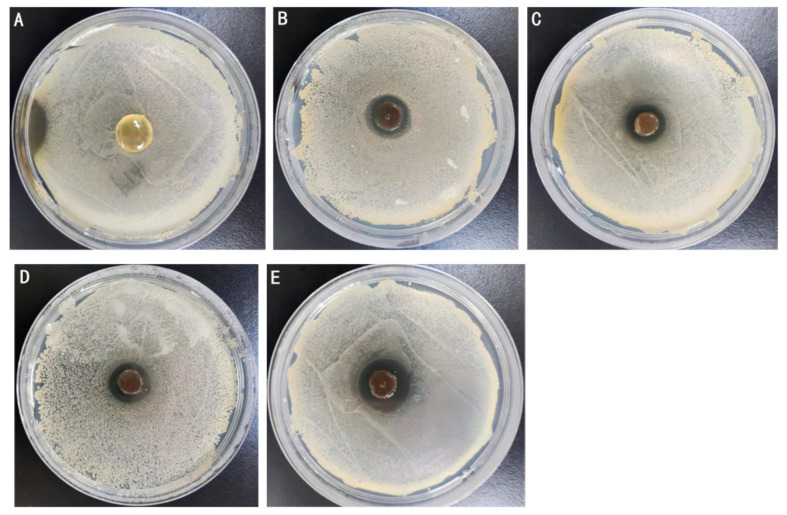
Inhibition test against *Staphylococcus aureus*. Note: Letters (**A**–**E**) correspond to test groups A~E, respectively.

Antibacterial capacity serves as an important quality parameter for medical dressings, with enhanced antibacterial properties directly correlating with improved clinical applicability. In this study, *Escherichia coli* (*E. coli*) and *Staphylococcus aureus* (*S. aureus*) were chosen to assess the antibacterial activity of various Ag-MOFs/OKC/PAM hydrogels using the antibacterial zone method (Figure 7 and Figure 8), where larger inhibition zones indicate stronger antibacterial effects.

As shown in the two figures, the results demonstrated a dose-dependent antibacterial activity. All four groups containing Ag-MOFs (B~E) exhibited distinct inhibition zones, and the zone diameter increased significantly with the increase in the Ag-MOFs content. The control group A, which contained no Ag-MOFs, showed no detectable antibacterial activity. Specifically, the inhibition zone diameters against *E. coli* were 26.8 mm (B), 29.8 mm (C), 31.7 mm (D), and 33.0 mm (E), while those against *S. aureus* were 14.5 mm (B), 15.8 mm (C), 17.2 mm (D), and 20.2 mm (E). These results indicated that the four Ag-MOFs/OKC/PAM hydrogels (groups B~E) exhibited varying degrees of antibacterial activity on both *E. coli* and *S. aureus*, with the antibacterial effect strengthening as the Ag-MOFs content increased. Among them, group E, which contained the highest Ag-MOFs content, displayed the strongest antibacterial performance. This enhanced antibacterial effect is likely attributed to the release of Ag^+^ from the Ag-MOFs, which possess broad-spectrum antibacterial properties. The higher the Ag^+^ content, the stronger the bactericidal ability [14,15].

By comparing the inhibition zone diameter of the same hydrogel against these two types of bacteria, it was observed that the antibacterial activity of the Ag-MOFs/OKC/PAM hydrogels against *E. coli* was stronger than against *S. aureus*. This suggests that the Ag-MOFs/OKC/PAM hydrogels exhibited significantly greater efficacy against Gram-negative bacteria than Gram-positive bacteria.

### 2.5. Cytotoxicity

**Figure 9:**
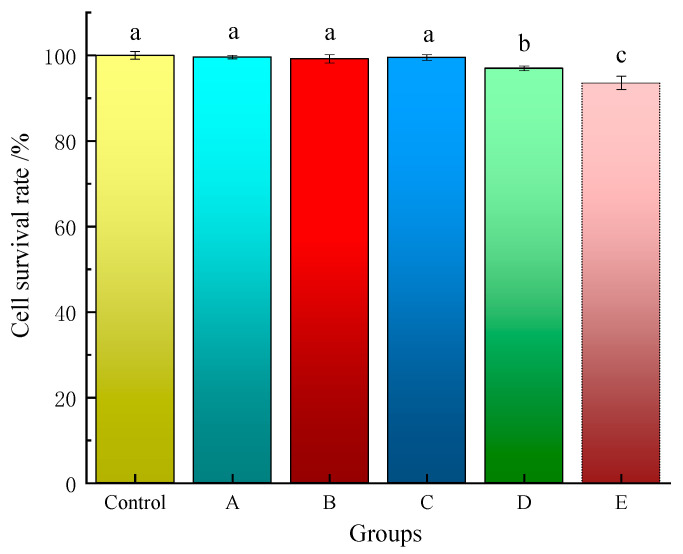
Toxicity of different hydrogels to L929 cells. Note: Different lowercase letters (a~c) above bars denote significant differences among groups (*p* < 0.05). Letters A~E correspond to test groups A~E, respectively.

The biocompatibility of medical dressings is crucial for wound healing, as it directly impacts the safety of the surrounding tissue. While silver-ion-based antibacterial agents are effective against bacteria, they may also exert certain toxic side effects on cells, implying that hydrogel dressings containing silver ions may exhibit some degree of toxicity. Therefore, it is essential to assess the potential cellular damage caused by such hydrogels [16,17]. To evaluate the cytotoxicity of the Ag-MOFs/OKC/PAM hydrogels and determine their suitability as medical dressings, L929 cells were used in this study. The results are shown in Figure 9.

Overall, after 48 h of exposure to the extracts of the composite hydrogel samples, the cell survival rates for all five hydrogels with varying Ag-MOFs contents exceeded 90%, consistent with the control group. This indicates that all five Ag-MOFs/OKC/PAM hydrogels exhibited excellent biocompatibility.

Among them, groups A~C showed exceptional biocompatibility, with all cell survival rates above 99%, closely matching the control group. No significant difference (*p* > 0.05) was observed between the control group and groups A~C. However, the cell survival rates of groups D and E were significantly lower than that of the control group (*p* < 0.05), at 96.96% (D) and 93.58% (E), respectively. These results suggest that the addition of low doses of Ag-MOFs had minimal impact on the survival rate of cells, implying that Ag-MOFs/OKC/PAM hydrogels with appropriately optimized Ag-MOFs contents demonstrate excellent biosafety profiles. A threshold effect appeared between 18~36 mg Ag-MOFs incorporation (groups D and E). Excessive Ag^+^ release from higher Ag-MOFs loadings may induce slight cellular damage [18]. Overall, although groups D and E exhibited mild cytotoxicity, their maintained cell viability above 90% still confirmed their excellent biocompatibility overall.

## 3. Conclusions

Hydrogels play a vital role in tissue engineering and regenerative medicine due to their 3D structure, excellent permeability, and superior drug delivery properties. As an algal polysaccharide, κ-carrageenan is an ideal hydrogel material due to its biocompatibility, abundant availability, and low cost. Although it lacks inherent bactericidal properties like most natural polysaccharides, it serves as an excellent carrier for MOFs. By integrating the sustained silver ion release from Ag-MOFs with the intrinsic hydrogel properties of κ-carrageenan, this study demonstrates the potential of Ag-MOFs/κ-carrageenan composites as high-performance antibacterial hydrogel wound dressings. The optimized dressings exhibited superior biological safety, minimal cytotoxicity, potent antibacterial properties, and significant wound-healing efficacy.

In this study, five OKC-based composite hydrogels (Ag-MOFs/OKC/PAM) were prepared with varying Ag-MOFs contents, ranging from 0 mg (group A) to 60 mg (group E). Through a comprehensive characterization of their physicochemical properties, antibacterial activity, and biocompatibility, including MC, WS, SR, TS, EB, and microstructure, the following universal advantages were identified across all hydrogels: (1) high moisture retention (MC exceeding normal skin level, providing an optimal moist environment for wound healing); (2) excellent water absorption capacity (equilibrium SR over 400%, enabling effective clearance of wound exudate); and (3) superior biocompatibility (cell survival rates exceeding 90%, suitable for clinical applications). These results confirm the significant potential of Ag-MOFs/OKC/PAM hydrogels as advanced wound dressings.

However, Ag-MOFs incorporation also produced concentration-dependent effects. For example, higher Ag-MOFs loading led to increased collapsing of the 3D pore structures, enhanced water solubility, reduced water resistance, and decreased SR and mechanical properties. In contrast, lower Ag-MOFs loading showed minimal microstructure impact, maintained water resistance, and improved mechanical properties, as demonstrated in group B. These results highlight the crucial influence of the Ag-MOFs concentration on the performance of Ag-MOFs/OKC/PAM hydrogels for medical applications. Further detailed research is necessary to optimize their formulation, analyze the formation process of their spatial structure, and study the antibacterial mechanism by which Ag^+^ is released from Ag-MOFs in order to maximize their clinical potential as next-generation wound dressings and minimize their toxic side effects.

## 4. Materials and Methods

### 4.1. Materials

K-carrageenan (KC) was purchased from Aladdin Chemistry Co., Ltd. (Shanghai, China). Acrylamide (AM), 2,2,6,6-tetramethylpiperidine-1-oxyradical (TEMPO), sodium hypochlorite (NaClO), sodium bromide (NaBr), N,N,N′,N′-tetramethylethylenediamine (TEMED), silver oxide (Ag_2_O), 1,4-cyclohexanecarboxylic acid (CHDA), and 1,3,5-triaza-7-phosphaadamantane (PTA) were purchased from Shanghai Yuanye Bio-Technology Co., Ltd. (Shanghai, China). All reagents were analytically pure. Mouse fibroblast cells (L929) were purchased from iCell Bioscience Inc. (Shanghai, China).

### 4.2. Methods

#### 4.2.1. Preparation of Oxidized κ-Carrageenan

**Figure 10:**
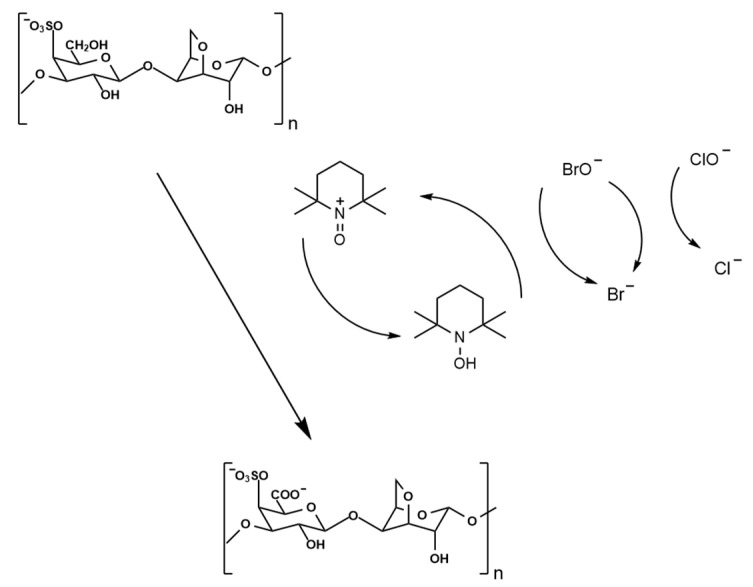
Schematic illustration of κ-carrageenan oxidation.

The preparation of oxidized κ-carrageenan (OKC) was carried out based on the method described by Cosenza et al. [19], with slight modifications (shown in Figure 10). Briefly, 7.9 g of κ-carrageenan powder was gradually added to 600 mL of deionized water heated to 90 °C in a water bath under constant stirring until complete dissolution was achieved. The solution was then cooled to room temperature, followed by the addition of 120 mg of TEMPO and 1.5 g of NaBr. After thorough mixing, 32.5 mL of NaClO solution (1.53 mol/L) was divided into five portions and added to the reaction system sequentially over 20 min. The pH value of the reaction system was adjusted to 10.5~10.8 using 1 mol/L NaOH solution after each addition of NaClO. The reaction was allowed to proceed for 100 min after the final addition of NaClO and was terminated by adding 250 mL of anhydrous ethanol. Then, the reaction solution was left to stand for 1 h, neutralized with 1 mol/L HCl, and concentrated to approximately 300 mL at 60 °C using a rotary evaporator. The concentrated solution was transferred to a dialysis bag and dialyzed against deionized water at 4 °C for 3 days, with the water replaced four times daily. Finally, the OKC was obtained through vacuum freeze-drying and stored at −20 °C. The oxidation rate of OKC was measured to be 74.5% according to the measurement method of Li et al. [11]

#### 4.2.2. Preparation and Characterization of Ag-MOFs

The preparation method of Jaros et al. [20] was followed, with slight modifications. Under constant stirring, 28 mL of methanol and 12 mL of deionized water, 92 mg of Ag_2_O, 172.4 mg of CHDA, and 125.6 mg of PTA were added into a beaker in turn. Next, the beaker was tightly packed with tin foil, and the mixture was stirred at room temperature for 30 min to react in the dark, after which a white turbid liquid was obtained. Then, 1.6 mL of ammonia water (1 moL/L) was used to dissolve the precipitate (pH 9.0). Finally, the solution was filtered and the filtrate collected into a crystallizing dish to crystallize it in a well-ventilated fume hood for 2 days to obtain Ag-MOFs, a light-yellow solid powder, which was stored at 4 °C.

The Ag-MOFs powder was scanned using a D8 ADVANCE X-ray diffractometer (Bruker, Stuttgart, Germany) with Cu-Kα radiation (λ = 1.54056 Å) in the range of 5° to 50° (2θ). Its molecular structure was characterized by Fourier transform infrared spectroscopy (FTIR) using an IRAffinity-1 spectrometer (Shimadzu, Kyoto, Japan) through pressing the dried sample into translucent pellets mixed with KBr. The spectral range was 4000~400 cm^−1^, with a resolution ratio of 4 cm^−1^.

#### 4.2.3. Preparation of Ag-MOFs/OKC/PAM Hydrogels

**Figure 11:**
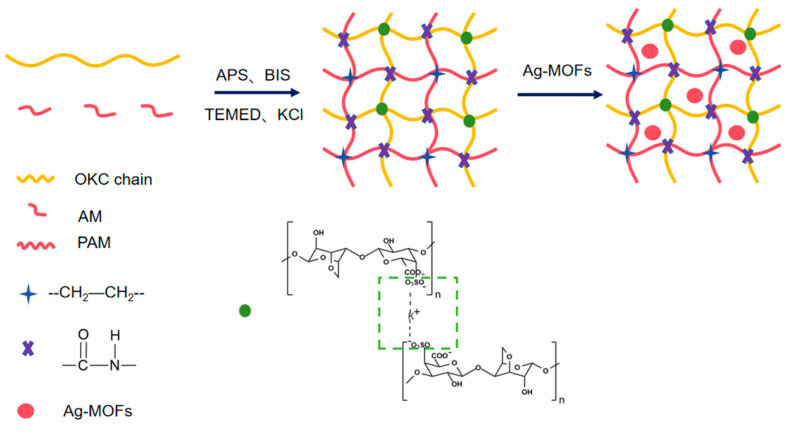
Process flowchart for Ag-MOFs/OKC/PAM hydrogel synthesis.

Five groups of OKC-based composite hydrogels loaded with different amounts of Ag-MOFs (Ag-MOFs/OKC/PAM), labeled as A to E, were prepared according to the methods described by Deng et al. [21] and Li et al. [11] (shown in Figure 11). In brief, 25 mL of deionized water was added to each of the five beakers and heated to 60 °C on a magnetic stirrer. Subsequently, 2.25 g of OKC was then added and stirred until completely dissolved, followed by the addition and dissolution of 3 g of AM. Different quantities of Ag-MOFs (0 mg, 12 mg, 24 mg, 36 mg, and 60 mg) were then introduced into the respective mixtures, and stirring continued for 20 min. Next, 900 μL of 1% BIS solution (mass volume ratio, g/mL), 20 μL of TEMED, 320 μL of APS (0.25 moL/L), and 60 mg of KCl were added, and the mixture was stirred for an additional 20 min. The reaction liquid was then poured into an acrylic mold measuring 90 mm × 90 mm × 1.5 mm, sealed, and allowed to react in a 60 °C incubator for 4 h to form the hydrogel samples. To minimize water evaporation, the beaker was sealed with plastic wrap during the dissolution and reaction processes. The specific compositions of each hydrogel are detailed in Table 1.

#### 4.2.4. Physicochemical Properties Analysis of Ag-MOFs/OKC/PAM Hydrogels

(1) MC and WS. The hydrogel samples were cut into small pieces of 15 mm × 15 mm. Each piece was weighed (*m*_0_), dried at 105 °C until a constant weight (*m*_1_) was achieved, and then immersed in 25 mL of PBS buffer (0.01 moL/L, pH 7.4) in a beaker. Next, the beaker was sealed with plastic wrap and placed in a 37 °C incubator for 24 h. Then, the pieces were removed and dried again at 105 °C to a constant weight (*m*_2_). MC and WS were calculated according to the following equations, respectively:

MC = (*m*_0_ − *m*_1_)/*m*_0_ × 100 (1)

WS = (*m*_0_ − *m*_2_)/*m*_0_ × 100 (2)

where MC is the moisture content (%), WS is the water solubility (%), *m*_0_ is the initial weight (g) of the hydrogel pieces, *m*_1_ is the weight (g) after the first drying, and *m*_2_ is the weight (g) after the second drying.

(2) SR. Following the method described by Zhang et al. [22], the hydrogel samples were cut into small pieces of 15 mm × 15 mm, weighed (*m*_0_), and immersed in 25 mL of PBS buffer (0.01 mol/L, pH 7.4) for the following specific time intervals: 1 h, 3 h, 6 h, 10 h, 18 h, 34 h, and 50 h. At each designated time point, the pieces were removed, surface moisture was carefully wiped off, and the pieces were weighed (*m*_t_) again. After weighing, the pieces were returned to their original containers to continue soaking. SR was calculated as follows:

SR = (*m*_t_ − *m*_0_)/*m*_0_ × 100 (3)

where SR is the swelling ratio (%), *m*_t_ is the weight (g) of the swollen hydrogel pieces after immersion for t h, and *m*_0_ is the initial weight (g) of the hydrogel pieces.

(3) Mechanical properties. According to the method of Zhai et al. [23], TS and EB of the hydrogels were measured using an electronic tensile testing machine (Dongri STC-50 KG, Dongguan, China) at ambient temperature after the hydrogel samples were cut into strips measuring 40 mm × 10 mm. The initial distance between the fixtures was set to 20 mm, and the stretching velocity was maintained at 50 mm/min. The mechanical properties were calculated as follows:

TS = *F*/(*a* × *D*) × 100 (4)

EB = *L*/*L*_0_ × 100 (5)

where TS is the tensile strength (MPa), *F* is the maximum tensile force (N), *a* and *D* are the width and thickness of the hydrogel strip (mm), respectively, EB is the elongation at break (%), *L* is the extension of the hydrogel strip at the point of breakage (mm), and *L*_0_ is the initial test distance (mm).

#### 4.2.5. Scanning Electron Microscopy Analysis of Ag-MOFs/OKC/PAM Hydrogels

After undergoing vacuum freeze-drying for 3 days, the Ag-MOFs/OKC/PAM hydrogels were immersed in liquid nitrogen to induce brittle fracturing. To observe the microstructure, the hydrogels were coated with gold and analyzed using scanning electron microscopy (SEM; Tescan MIRA LMS, Brno, Czech Republic) at an accelerating voltage of 15.0 kV.

#### 4.2.6. Antimicrobial Activity of Ag-MOFs/OKC/PAM Hydrogels

The antibacterial properties of the hydrogels against *Escherichia coli* and *Staphylococcus aureus* were evaluated using the agar diffusion plate method, following the Chinese National Standard GB/T 20944.1-2007 [24]. Briefly, LB nutrient agar plates were uniformly coated with bacterial suspension. The hydrogel samples were cut into 9 mm diameter rounds and placed at the center of the agar plate. After culturing the plates at 37 °C for 24 h, the diameters of the inhibition zones were measured.

#### 4.2.7. Cytotoxicity Test of Ag-MOFs/OKC/PAM Hydrogels

The biocompatibility of the Ag-MOFs/OKC/PAM hydrogels was evaluated using a Cell Counting Kit-8 (CCK-8) assay with L929 cells.

Sample Preparation: Different composite hydrogels were lyophilized and then leached with culture medium for 24 h at a concentration of 1 mg/mL (hydrogel sample/culture medium).

Cell Culture Preparation: L929 cells, previously frozen in liquid nitrogen, were rapidly thawed, transferred to a 15 mL sterile centrifuge tube containing 7 mL of complete medium, and then centrifuged at 1000 rpm for 5 min (at 25 °C). The resulting cell pellet was transferred to a 25 T cell culture flask with 5 mL of medium supplemented with 10% fetal bovine serum (FBS) and incubated at 37 °C for 8 h in a 7.5% CO_2_ atmosphere. After aspirating the medium, 5 mL of fresh medium containing 10% FBS was added, and the cells were further incubated until they reached full confluency.

Cell Seeding and Treatment: Before seeding, the culture medium was sucked dry, and the cells were washed with PBS and then digested with 0.25% trypsin. After the trypsin was removed, the cells were cultured with complete medium containing 10% FBS, counted using a cell counter, diluted with complete culture medium, seeded onto a 96-well plate with a density of 3000 cells per well, and cultured in an incubator at 37 °C for 24 h under a 5% CO_2_ atmosphere. Then, the culture medium was replaced with fresh medium containing different sample leaching solutions, and the cells were cultured for an additional 48 h.

CCK-8 Assay: After incubation, the medium in each well was aspirated, and the cells were rinsed three times with PBS. Subsequently, 100 µL of culture medium and 10 µL of CCK-8 solution were added to each well, followed by incubation for another 2 h. The absorbance at 450 nm was measured using a microplate reader. The control group consisted of cells cultured in medium without sample leaching solutions, while the blank group contained medium and CCK-8 solution without cells. The formula for calculating cell survival rate is as follows:

Cell survival rate (%) = (A_sample_ − A_blank_) ÷ (A_control_ − A_blank_) × 100 (6)

where A_sample_ is the absorbance of the sample group, A_blank_ is the absorbance of the blank group, and A_control_ is the absorbance of the control group.

#### 4.2.8. Data Analysis

Three parallel tests were conducted for each kind of hydrogel under the same conditions to obtain the average values. Data were statistically analyzed using OriginPro 2022. Significant differences were assessed using one-way analysis of variance (ANOVA), with *p*-values < 0.05 considered statistically significant.

## Figures and Tables

**Table 1:** Compositions of the OKC-based composite hydrogels with different contents of Ag-MOFs.

Samples	Ag-MOFs/mg	OKC/g	AM/g	BIS/μL	TEMED/μL	APS/μL	KCl/mg	H_2_O/mL
A	0	2.25	3	900	20	320	60	25
B	12	2.25	3	900	20	320	60	25
C	24	2.25	3	900	20	320	60	25
D	36	2.25	3	900	20	320	60	25
E	60	2.25	3	900	20	320	60	25

## Data Availability

Data will be made available upon request.
